# Early-Life Sublethal Thiacloprid Exposure to Honey Bee Larvae: Enduring Effects on Adult Bee Cognitive Abilities

**DOI:** 10.3390/toxics12010018

**Published:** 2023-12-23

**Authors:** Xiasang Chen, Airui Li, Linghong Yin, Li Ke, Pingli Dai, Yong-Jun Liu

**Affiliations:** State Key Laboratory of Resource Insects, Institute of Apicultural Research, Chinese Academy of Agricultural Sciences, Beijing 100193, China

**Keywords:** honey bee larvae, thiacloprid, learning and memory, neuron apoptosis, transcriptomic

## Abstract

Honey bees have significant ecological and economic value as important pollinators, but they are continuously exposed to various environmental stressors, including insecticides, which can impair their health and cause colony decline. (1) Background: Cognitive abilities are vital for the functional maintenance of honey bees; however, it remains unknown if chronic, low-dose exposure to thiacloprid during the larval stage impairs the cognitive abilities of emerged adult honey bees. (2) Methods: To explore this question, honey bee larvae were fed 0, 0.5, and 1.0 mg/L thiacloprid during their developmental phase. Then, the cognitive (i.e., olfactory learning and memory) abilities of adult honey bees were quantified to assess the delayed impacts of early-stage thiacloprid exposure on adult honey bee cognition. Neural apoptosis and transcriptomic level were also evaluated to explore the neurological mechanisms underlying these effects. (3) Results: Our results revealed that chronic larval exposure to sublethal thiacloprid impaired the learning and memory abilities of adult honey bees by inducing neuronal apoptosis and transcriptomic alterations. (4) Conclusions: We highlighted a previously unknown impairment caused by thiacloprid in honey bees.

## 1. Introduction

Honey bees (*Apis mellifera* L.), the most widespread bee species of the family *Apidae*, show excellent performance in pollination services and maintaining ecosystem diversity. In recent years, annual losses in honey bee populations have been increasing, which can be attributed to multiple factors, including viruses, bacteria, fungi [1], parasites [2], fungicides [3], and poor nutrition [4]. Pesticides, especially neonicotinoids, are a major challenge to the well-being of honey bees and bee colonies [5].

Neonicotinoids are widely used in agriculture, especially in sucking and chewing pest control because of their remarkable efficacy and lower toxicity as compared to conventional pesticides [6]. However, neonicotinoids can be found throughout the entire plant due to their systemic properties [7]. Prior research has found neonicotinoid residues within honey or pollen samples [8,9] as well as in the matrices in the hive [10], emphasizing the possible adverse consequences of these pesticides on honey bees [5]. 

Thiacloprid, a cyano-substituted neonicotinoid, has been shown in previous studies to exhibit relatively lower toxicity toward honey bees in comparison to other nitro-substituted neonicotinoids, such as thiamethoxam and acetamiprid [11,12]. However, numerous studies have demonstrated it also had negative effects on honey bee health. Thiacloprid has been found to disrupt honey bee physiological homeostasis [12], decrease survival rates in honey bee colonies, compromise immunocompetence [13], disrupt metabolism [14], and induce transcriptional alterations in the brain of honey bee [15]. Furthermore, thiacloprid exposure has been shown to disturb foraging and homing behaviors [16] as well as cognitive performance [17] of honey bees. While previous investigations have mostly concentrated on the harmful toxic impacts of thiacloprid on adult honey bees, it is worth mentioning that honey bees can transport thiacloprid to the hive through the collection of nectar or pollen during foraging. Due to thiacloprid’s longer dissipation half-life and system activity, the impacts of thiacloprid on honey bees may persist for prolonged periods [18]. All the honey bees within the hive, particularly the highly sensitive larvae, are vulnerable to neonicotinoid exposure, which leads to reproductive or developmental problems of the colonies. Studies have shown that being exposed to neonicotinoids resulted in decreased bee reproductive capacity [19], delayed honey bee development [20], increased solitary honey bee mortality and decreased cocoon weight [21], and altered ultrastructure of honey bee organs and cellular function [22]. Learning acquisition and memory retention are vital for foraging success and communication with mates in honey bees. However, the consequences of being exposed to thiacloprid on adult honey bee behaviors subsequent to larval stage exposure are rarely shown. Furthermore, the neurological mechanisms underlying these impairments have not been extensively explored.

Our study aimed to assess the response of adult honey bees to thiacloprid contamination in their early larval stage, even though the toxicity of thiacloprid has been extensively studied in adult bees. We provided evidence supporting the chronic toxicity of thiacloprid in honey bees [14,23,24]. Exposure to thiacloprid during the larval stage resulted in reduced survival rates and body weight. Furthermore, larval exposure to thiacloprid impaired the learning and memory abilities of adult bees. Additionally, thiacloprid induced neural apoptosis and transcriptome alterations in honey bees. Moreover, our results shed light on the impact of thiacloprid on sustainable honey bee survival and underscore the importance of safe pesticide use. 

## 2. Materials and Methods

### 2.1. Chemical Materials

Three concentrations of thiacloprid were prepared for exposure experiments: 0 mg/L (TH0), 0.5 mg/L (TH0.5), and 1.0 mg/L (TH1.0). The thiacloprid stock solution was 250 mg/L. To make the stock solution, 10 mg thiacloprid (Sigma-Aldrich, St. Louis, MO, USA) was dissolved in 1 mL dimethyl sulfoxide (DMSO) and subsequently diluted with distilled water. Food was freshly prepared before the experiment, following the feeding requirements for bee larvae at different developmental stages, for details, refer to Table S1 [25]. In the thiacloprid treatment groups, freshly prepared larvae food was used to dilute thiacloprid to TH0.5 and TH1.0. The concentrations of thiacloprid are significantly lower than the LC_50_ value tested in our previous study [26] and within the range of thiacloprid found in the field [13,27]. Prior investigations have indicated that 0.1% dimethyl sulfoxide (DMSO) does not significantly affect the mortality and development of honey bee larvae or their olfactory associative behavior [28]. The maximum DMSO concentration used in our thiacloprid exposure dose was 0.01%, which should not affect the physiological status of honey bees. Additionally, our previous results [24] showed that 0.02% DMSO had no impact on cell apoptosis. Therefore, we did not include a solvent control (DMSO group) in this study. 

### 2.2. Sample Preparation 

Honey bees (*Apis mellifera* L.) were acquired from the Institute of Apicultural Research apiary (39°59′35″ N, 116°11′59″ E), Chinese Academy of Agricultural Science. Prior management measures were implemented for maintaining the relative health of honey bees, including the detection of both mite infestation and common viral infections (Israeli acute paralysis virus, black queen cell virus, deformed wing Virus, etc.). The bees underwent visual inspection at mite-free status. For the purpose of collecting emerged honey bees at the same hatching age, three different hives of bees with blank brood frames were provided for three healthy honey bee queens to oviposit on day one. On the fourth day, healthy bee larvae from each brood frame were transferred to 48-well plates for laboratory larvae rearing, and this day was designated as day one (D1). See Table S1 in the Supplemental Materials for food formulas and amounts supplied from D1 to D6 for rearing honey bee larvae. During this period, three different experimental groups (TH0, TH0.5, and TH1.0) were exposed to thiacloprid, with three biological replicates of larvae selected from three distinct brood frames. The bee larva plates were incubated in an artificial incubator (RXZ-380C, Ningbo, China) at 35 ± 1 °C and 95% humidity. Thiacloprid exposure was administered for a total of 6 days (D1–D6).

**Figure 1:**
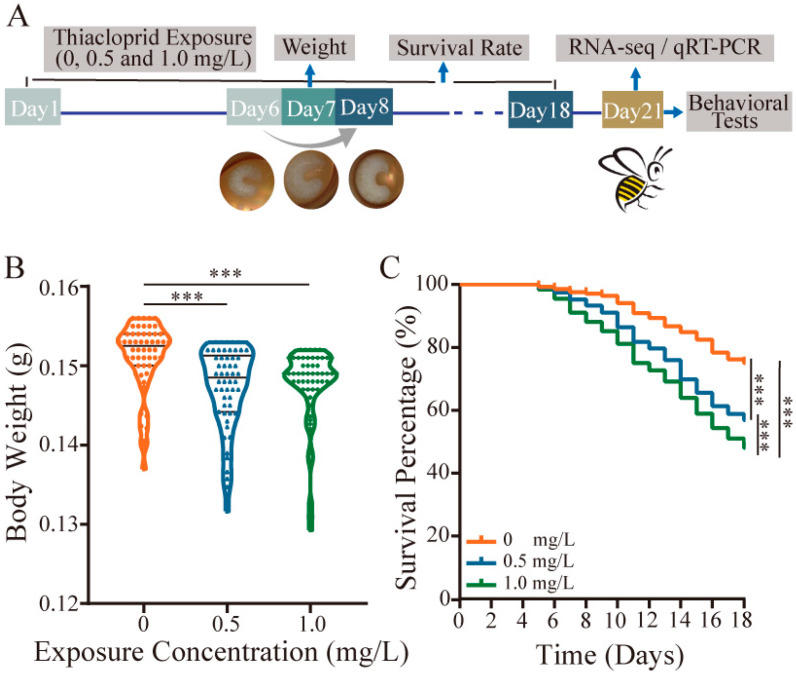
Negative effect of thiacloprid on honey bee body weight and survival rate. (**A**) Schematic diagram of the experimental setup. Blue arrows delineate diverse tests throughout the experimental process, while black lines demarcate the time intervals used for survival rate calculations. The gray arrows symbolize the morphological transformation of honeybee larvae from D6 to D8. Exposure of the larval stage to thiacloprid resulted in a significant reduction in body weight (**B**) and poor survival rate (**C**) of honey bees compared with control. *** *p* < 0.001.

From the 7th day (D7), the honey bee larvae were moved to 24-well plates where they transitioned into the pupal stage, a non-feeding, transformative phase while in the pupal stage, pupae rely on energy reserves accumulated during their larval stage and are not actively fed until the emergence of the bees. Throughout the pupal stage, the conditions within the incubator were maintained at 35 ± 1 °C and 75% relative humidity. From D8 to D18, the larvae survival rate was recorded daily. Additionally, on the 7th day before transfer, we measured the weight of each honey bee larvae. After honey bee emerged, they were collected and reared in a wooden cage, exclusively fed on 50% sucrose (*w*/*w*) without proteins or microbiome bacteria to minimize potential confounding factors in the incubator for further testing. A schematic diagram of the experimental setup for bee samples at various time points in this study is depicted in Figure 1A.

### 2.3. Behavioral Experiments 

In the foraging and homing of honey bees, learning and memory play a crucial role. Proboscis extension reflex (PER) was employed as a behavioral indicator of honey bee sucrose acuity and olfactory associative learning and memory.

Responses to sucrose: In total, 60 newly emerged honey bees on day 19 (D19) from each group were randomly collected to conduct honey bee response to sucrose tests. Six sucrose concentrations (1%, 5%, 10%, 25%, 30%, and 50%) were used. Before the tests, honey bees were starved for 2 h. Then, the head of each bee was secured in a small tube while allowing the rest of its body to move freely. Briefly, six 1 mL syringes filled with sucrose solution in order of sucrose concentrations from low to high were placed on the honey bee antennae for 3 s, respectively, and the PER response was recorded for each bee. A three-minute interval was provided between each presentation. 

Associative olfactory learning and memory assays: A total of 68–76 honey bees per treatment group were used to evaluate their learning and memory performances on Day 21, which is the time when the honey bee’s olfactory system has fully developed [29]. We applied hexanal (66-25-1, Sigma, St. Louis, MO, USA) as a conditioned stimulus (CS+) paired with a 30% sugar stimulus (unconditioned stimulus, US), and R-limonene, which served as the other conditioned stimulus (CS-) (95327-98-3, Sigma, St. Louis, MO, USA), was not paired with any sucrose. Hexanal and Limonene were diluted to 10% with paraffin oil, and 10 µL of diluted solutions was dropped onto 0.5 cm × 1 cm filter paper, which was then placed on a Pasteur pipette and connected to the gas stream tube. The tests comprised two parts: a learning acquisition phase and a memory retrieval phase. When the training started, a gas stream (flow rate of 5 mL/s) containing limonene or hexanal was opened. For the paired conditioned stimulus, honey bees were given Hexanal odor stimulation for 6 s close to their antenna, and 10 µL of sucrose at 30% as a reward was given to honey bees for the last 3 s. For the unpaired conditioned stimulus, honey bees were only given limonene for 6 s without a sucrose reward. The learning acquisition training was repeated five times. After finishing the training, memory recalls were carried out using the same odorants used for training but without any rewards after 10 min, 3–12 h, and 24 h of training, which correspond to short-term (10 min), middle-term (3 h, 6 h, and 12 h), and long-term (24 h) memory, respectively. The percentage of PER in each treatment group under paired and unpaired conditioned stimuli was quantified to identify learning and memory abilities. In addition, the acquisition score was calculated based on the frequency of each honey bee’s response to the conditioned odor during the five acquisition trials. The score ranged from 0 to 5 and was used to verify the success of bee-associative olfactory learning. The odor discrimination index (DI) measures memory recall ability by computing the differences in PER between paired conditioned odor hexanal and unpaired conditioned odor R-limonene for short-term memory (10 min), middle-term memory (3–12 h), and long-term memory (24 h). The DI has a range of −1 to 1, with positive values indicating that PER to hexanal is greater than PER to unpaired limonene, and vice versa.

### 2.4. Immunohistochemistry for Neural Apoptosis

In total, 5–8 honey bee brains were sectioned horizontally into 20 µm thick slices using a Leica microtome (Leica CM1950, Leica, Wetzlar, Germany) for immunofluorescence staining. Brain slices were processed in an immunohistochemical blocker (100 mL, Beyoncé, Beijing, China) with 0.1% Triton X for 10 min. Then, they were moved to 0.1 M Tris-HCL (pH = 7.4) at RT for 30 min. A freshly prepared mixture of 1:9 ratio of TUNEL reaction solution (A solution) to cobalt chloride hexahydrate (B solution) was incubated with brain slices for 2 h in a dark humidified box at 37 °C. Then, DAPI staining (D9542, Sigma, St. Louis, MO, USA) was performed for all sections. 

Brain cell apoptosis in each tested group was imaged using a confocal microscope (SP8, Leica, Wetzlar, Germany). Apoptotic cells in five areas of the bee brain were examined separately, including antennal lobes (AL), internal chiasma (IC), mushroom bodies (MB), outer optic chiasma (OC), and lamina (LA). Cell counting of apoptotic cells in each group was performed using Image J. The rate of neural apoptosis is the ratio of TUNEL-positive cells to the total cells.

### 2.5. RNA Extraction, Reverse Transcription, Quantitative qPCR, and Transcriptome Data Analysis

For RNA sequencing, each treatment group had three biological replicates, and RNA of each replicate was extracted from five honey bee brains. TRIzol reagent was employed for the extraction process following the manufacturer’s protocols. For qRT-PCR, the brain RNA was collected using the RNA extracted kit (DP451, Tiangen Biotech, Beijng, China). Quality control of RNA was examined using a Nanodrop 2000 (Thermo Scientific, Wilmington, DE, USA) and quantified using a Bioanalyser (Agilent 2100, Santa Clara, CA, USA). For reverse transcription, the Prime Script™ RT Reagent Kit (RR047A, Takara Bio, Inc. Shiga, Japan) was used, and 1 μg qualified RNA was used for the reverse transcription process.

Quantitative(q)PCR was performed to identify differentially expressed genes (DEGs). We used β-actin as the endogenous control gene. Full-sequence information of whole primers can be found in Table S2 of the Supporting Information. Relative RNA quantification was performed using the comparative 2^−ΔΔCt^ method.

Libraries of 150 bp paired-end reads from each of the three quantified RNA samples, corresponding to different exposure doses, were constructed using the TruSeqTM RNA Preparation Kit (Illumina, San Diego, CA, USA) and sequenced using the Novaseq 6000 System (Illumina). Clean reads were mapped to the reference genome (https://www.ncbi.nlm.nih.gov/genome/?term=apismellifera, accessed on 17 September 2023) using HISAT (Version 2.1.0) software in the orientation mode. Differential expression genes (DEGs) across different treatment groups were calculated with RSEM (Version 1.3.3), and gene abundances were quantified using the transcripts per million reads (TPM) method. The comparative analysis of DEGs between different treatment groups was performed using DESeq2 (Version 1.24.0) with cutoffs of |log_2_FC| > 0.585 and a *p*-value < 0.05. Downstream analyses of DEGs, including Venn analysis, hierarchical clustering analysis, and principal component analysis, functional-enrichment (GO and KEGG) analyses, were further performed to illustrate the effect of thiacloprid exposure on honey bees.

### 2.6. Statistical Analysis

Shapiro–Wilk was used to evaluate data normality. Survival rate was evaluated using the Log-rank test. Body weight, neural apoptotic rates, and RT-qPCR results were assessed using one-way ANOVA and Tukey’s multiple comparisons. Behaviors results were assessed using Kruskal–Wallis and Dunn’s multiple comparisons tests. The level of statistical significance was less than 0.05. Differentially expressed gene (DEG) quantification was performed using DEGseq2 with |log_2_ FC | > 0.585 and *p* < 0.05. 

## 3. Results

### 3.1. Thiacloprid Affects Honey Bee Survival and Body Weight 

Thiacloprid was administered to the early larval stage for six days. On Day 7, the body weight of the larvae was measured. Survival rates were recorded for the entire duration of the experiment until Day 18. The results showed that TH0.5 and TH1.0 thiacloprid treatments at the larval stage remarkably reduced honey bee’s body weight compared with control on Day 7 (Figure 1B, *p* < 0.001). 

Simultaneously, we found a dosage-dependent impact of thiacloprid on the survival rate of honey bees from larvae to adulthood (Figure 1C). Larvae exposed to TH0.5 and TH1.0 of thiacloprid had a survival rate of less than 60% on Day 18 when the pupa transforms to emerged honey bees, which was significantly lower than control group (*p* < 0.001). Our results suggest that thiacloprid exposure posed a threat to honey bee growth and development by decreasing both body weight and survival throughout their developmental stages from larvae to adulthood. 

### 3.2. Thiacloprid Impaired Honey Bee Behaviors 

**Figure 2:**
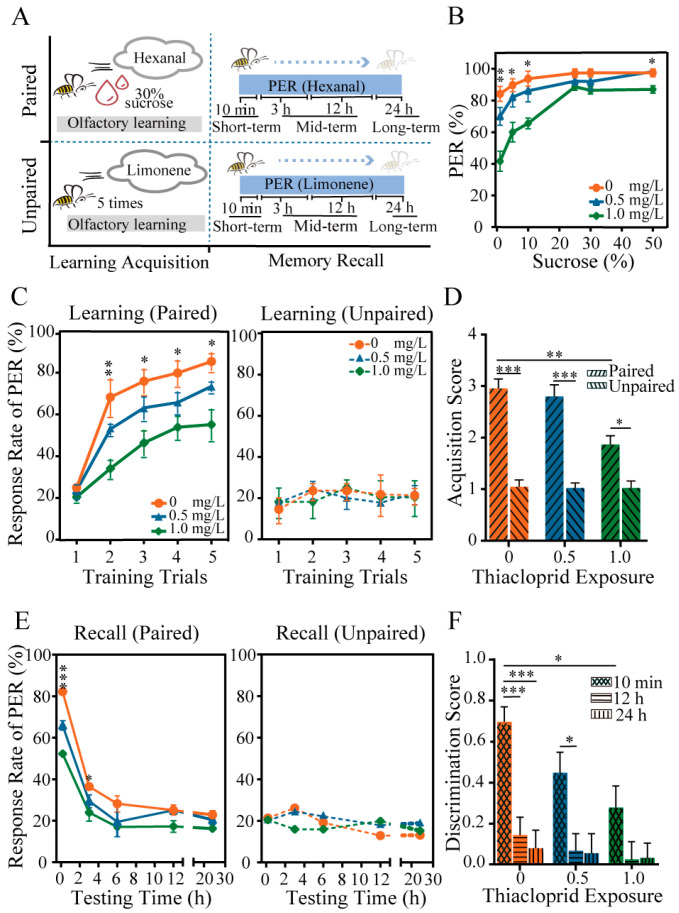
Impaired performance in honey bee response to sucrose, learning and memory of adult honey bees due to larvae thiacloprid exposure. (**A**) Schematic illustration of learning and memory tests. (**B**) Comparison of honey bee response to sucrose among thiacloprid-treated groups. (**C**) Olfactory learning acquisition and (**D**) scores of honey bees under paired and unpaired conditions with five training trials. (**E**) Summary of memory retention of honey bees under paired and unpaired conditions in each group. (**F**) Evaluation of odor discrimination score as a measure of memory retrieval ability in all experimental groups. * *p* < 0.05, ** *p* < 0.01, *** *p* < 0.001. Error bar represents mean ± SEM.

To assess the potential influence of thiacloprid exposure during the larval stage on behavioral changes of emerged honey bees, we evaluated honey bee response to sucrose and learning and memory ability, as illustrated in Figure 2A. The results presented in Figure 2B show the percentage of PER of emerged bees exposed to six different sucrose solutions (1%, 5%, 10%, 25%, 30%, and 50%) after larval exposure to thiacloprid at concentrations of TH0, TH0.5 and TH1.0. The PER rates of honey bees increased as the sucrose concentration increased across all groups. However, exposure to thiacloprid led to a significantly reduced PER of emerged bees to low sucrose concentrations (1%: *p* < 0.01; 5% and 10%: *p* < 0.05) (Figure 2B). We did not observe significant differences in PER between two thiacloprid-treated groups to sucrose concentrations greater than 25%. The findings suggest that chronic larval exposure to thiacloprid impaired honey bee response to sucrose in emerged bees, which may diminish their foraging abilities.

Next, the effects of thiacloprid on the cognitive abilities of adult honey bees were tested. In the learning acquisition session shown in Figure 2C, the PER rates of all treated honey bees trained with hexanal paired with sucrose increased as the training trials progressed. Moreover, as the dose of thiacloprid exposure to honey bees increased, the PER rates decreased significantly compared to the control in training trials 2–5 (trial 2: *p* < 0.01; trial 3–5: *p* < 0.05), and this effect was dose dependent. Conversely, there were no statistical differences in learning performance in unpaired conditions across training sessions (trials 1–5: *p* > 0.05), as demonstrated in the right panel of Figure 2C. We found that honey bees in paired conditions exhibited higher learning acquisition scores than those in unpaired conditions across all treatment groups, as depicted in Figure 2D. Moreover, under paired conditions, a significant decrease in acquisition scores in the TH1.0 group was observed compared with the control group (*p* < 0.01). 

The short-term (10 min), middle-term (3 h, 6 h, and 12 h), and long-term (24 h) memory recall of honey bees were examined by testing PER to hexanal and limonene odors (in Figure 2A). Honey bees treated with thiacloprid in their larval stage experienced memory decay in the paired training of hexanal with a sucrose reward compared to the unpaired training of limonene without a sucrose reward (Figure 2E,F). Furthermore, a decrease in the PER rates was found in the memory retrieval of honey bees under the paired condition, which was found to be consistent across all experimental groups in Figure 2E. Chronic thiacloprid exposure substantially impaired the short-term (10 min) and early middle-term (3 h) memory ability of adult honey bees in comparison to the controls (left panel of Figure 2E, 10 min: *p* < 0.001, 3 h: *p* < 0.05). In memory recall tests conducted 3 h after training, no significant difference in PER rates was observed across treatment groups for honey bees in the paired condition, as illustrated in the left panel of Figure 2E. Likewise, no significant difference was detected among all experimental groups across each time point of memory recall tests for the unpaired groups (Figure 2E, right panel).

Meanwhile, the discrimination score for hexanal and limonene was calculated for all treatment groups at 10 min, 12 h, and 24 h after learning acquisition training. The result in Figure 2F revealed that thiacloprid significantly reduced honey bee memory ability in odor discrimination (TH0 vs. TH1.0, *p* < 0.01). Moreover, honey bees in the TH0 and TH0.5 treatment groups exhibited significantly higher discrimination scores in short-term (10 min) memory recall than middle-term (12 h) or long-term (24 h) recall (10 min vs. 12 h or 24 h: *p* < 0.05, *p* < 0.001). 

### 3.3. Thiacloprid Exposure Caused Honey Bee Neuronal Apoptosis 

**Figure 3:**
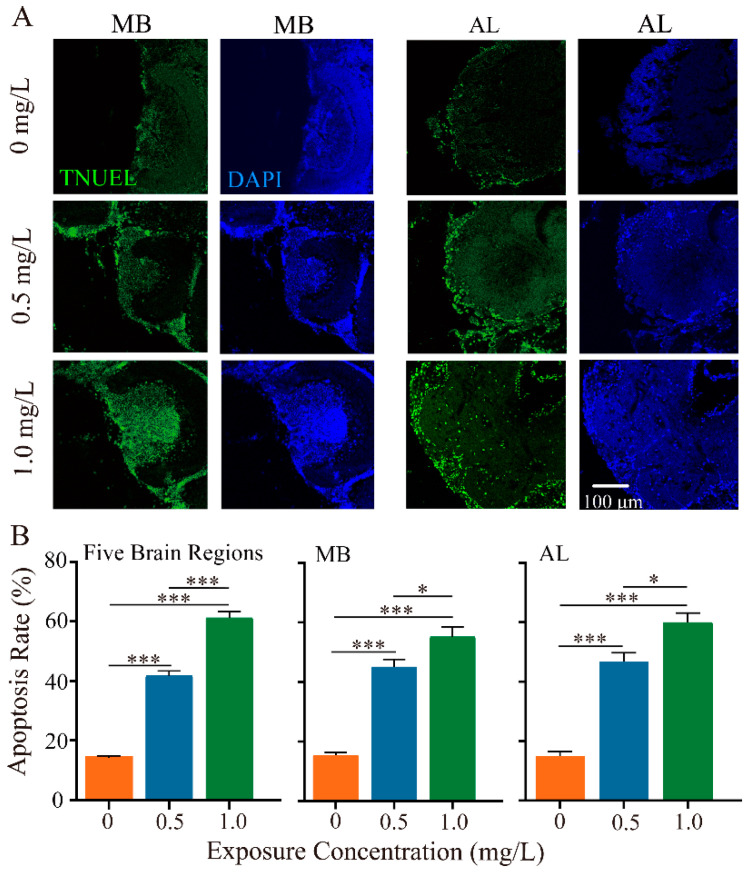
Thiacloprid induced apoptosis in mushroom bodies (MB) and antennal lobes (AL) of adult honey bees. (**A**) Representative images of cell apoptosis in the MB and AL regions using the TUNEL (TUNEL, green; DAPI, blue) assay in different thiacloprid-treated groups. (**B**) Quantitative assessment of apoptosis cells in the entire brain, MB, and AL, separately. Error bars represent mean ± SEM. * *p* < 0.05, *** *p* < 0.001.

Given the neurotoxicity of neonicotinoid pesticides on honey bees, we next investigated the impacts of thiacloprid on neuronal apoptosis of honey bee brains using the TUNEL assay. We examined neuron apoptosis in five brain regions, focusing specifically on the MB and AL brain regions, which play critical roles in olfactory coding, learning, and memory abilities. Figure 3A illustrates TUNEL-positive apoptotic neurons in the MB and AL regions at varying dosages of thiacloprid. Detailed apoptotic outcomes in the remaining three brain regions are provided in Figure S1. High concentration treatment group led to more apoptosis (more green-marked cells) in both MB and AL regions. In comparison to the control, neuronal apoptosis in MB and AL regions was significantly higher in the thiacloprid-exposed groups (Figure 3B, MB region: *p* < 0.001; AL region: *p* < 0.001). Specifically, the apoptosis rate in MB increased from 15.46% to 45.08% and 55.23% in the thiacloprid-treated groups (TH0.5 and TH1.0) compared to the controls. Similarly, the apoptosis rate in the AL region was also much higher in the thiacloprid treatment groups, with corresponding apoptosis rates in the AL region increasing to 46.75% and 59.94% for TH0.5 and TH1.0 treatments, respectively.

### 3.4. RNA-Seq Data Analysis of Honey Bees Exposed to Thiacloprid

**Figure 4:**
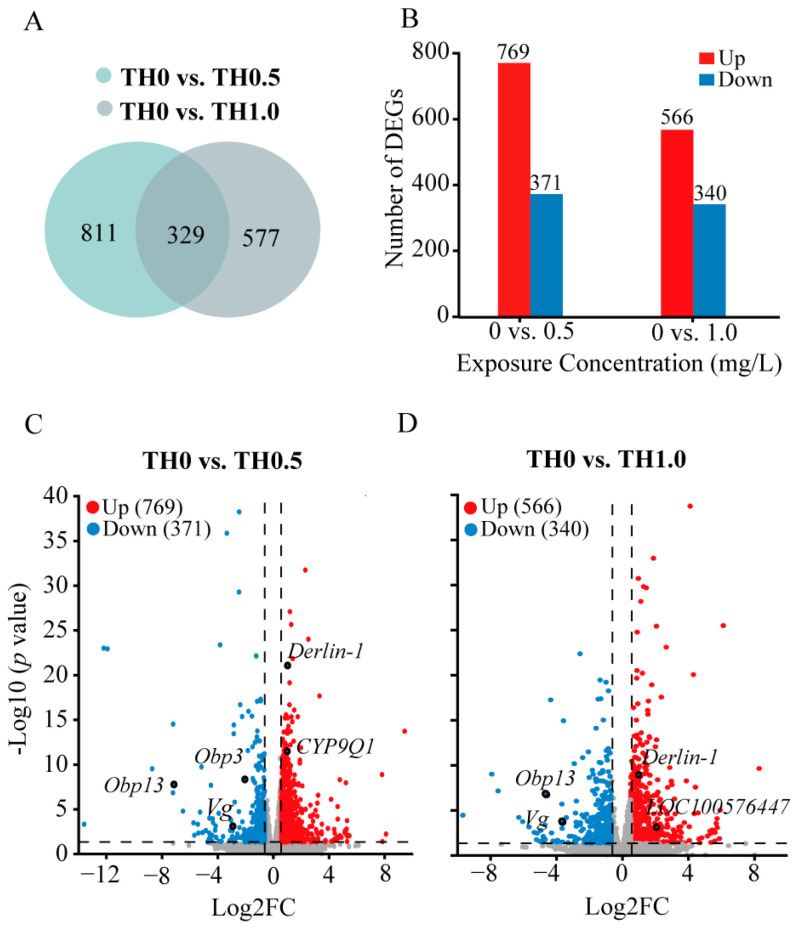
Distinct transcriptomic signatures in honey bees from the tested groups identified using differential expression genes (DEG) analysis. (**A**) Numbers of the DEGs of honey bees between thiacloprid and control groups (**B**) The number of upregulated genes and downregulated genes among the DEGs in the comparisons of TH0 vs. TH0.5 and TH0 vs. TH1.0. (**C**,**D**) Volcano plots illustrate the upregulated and downregulated DEGs in honey bees in the thiacloprid-treated groups compared to the control group.

To assess relevant genetic alterations induced by thiacloprid in honey bee brains, we performed transcriptome analysis on adult honey bees. Data were submitted to the NCBI Bioproject database (Bioproject ID: PRJNA929684). The screening thresholds of DEGs were set using corresponsive parameters (|Log_2_ FC| > 0.58 and *p <* 0.05). Venn diagrams in Figure 4A show the DEGs numbers between treatment groups. Of the 1140 DEGs detected in the thiacloprid-treated groups of TH0 vs. TH0.5, we obtained 769 upregulated genes and 371 downregulated genes. Similarly, of 906 DEGs in the thiacloprid-treated groups of TH0 vs. TH1.0, 566 upregulated genes and 340 downregulated genes were identified (Figure 4B). Principal component analysis and hierarchical clustering map results are shown in Figure S2. 

Furthermore, volcano plots were utilized to visualize DEGs among the experimental groups (Figure 4C,D). Red and blue points on the plots denoted genes with significantly increased or decreased expression in thiacloprid treatments. Noticeably, relative expression of *Obp3* and *Obp13* genes, which are two important odorant binding proteins in the honey bee brain olfactory system, were significantly downregulated in the thiacloprid-treated group. The downregulation of the odorant-related genes may lead to impaired odor perception in thiacloprid-exposed honey bees. In addition, a significant downregulation of the *Vg* gene, which has been linked to the protection of bees from oxidative damage, was noted in all thiacloprid treatment groups. *Derlin*-*1* and *LOC100576447* genes associated with cell apoptosis are upregulated in response to thiacloprid treatment; this finding is consistent with the observed increase in apoptosis rates in honey bee brain neurons in Figure 3. Moreover, the *CYP9Q1* gene that is involved in detoxification was upregulated in the TH0.5 thiacloprid group compared to control (Figure 4C). 

In summary, DEG analysis revealed significant changes in the gene expressions of adult honey bees who were exposed to thiacloprid during their larval stage, indicating a potential impact on their olfactory process and individual health. 

### 3.5. Functional Analysis of DEGs

**Figure 5:**
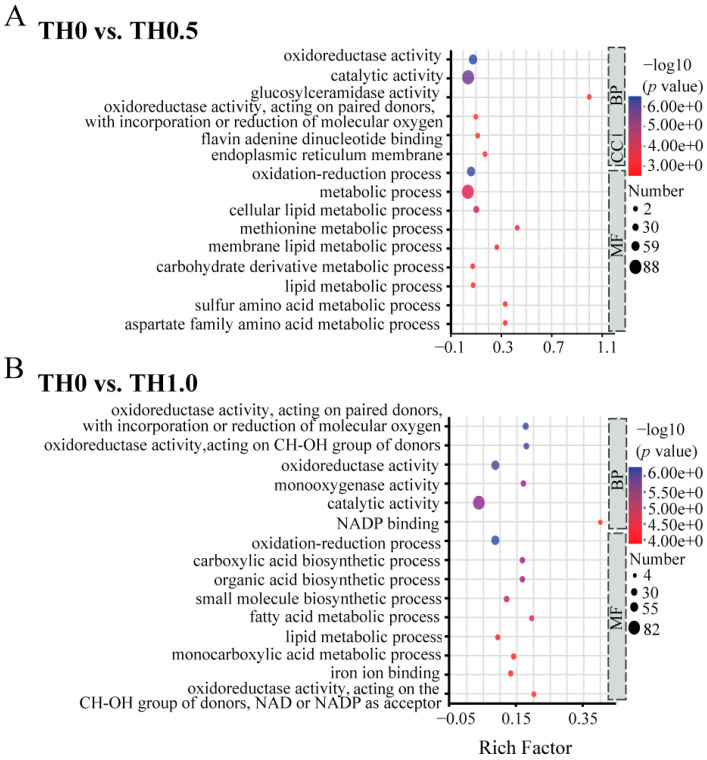
GO functional enrichment analysis of DEGs in honey bees across different treatment groups. (**A**) GO down-enrichment analysis of DEGs in the low concentration group (TH0 vs. TH0.5) and (**B**) the high concentration group (TH0 vs. TH1.0). The vertical axis represents the secondary classification terms of GO, while the horizontal axis represents the Rich factor. The size and color of the dots correspond to the number of genes and the −log10 (*p* value) ranges, respectively.

**Figure 6:**
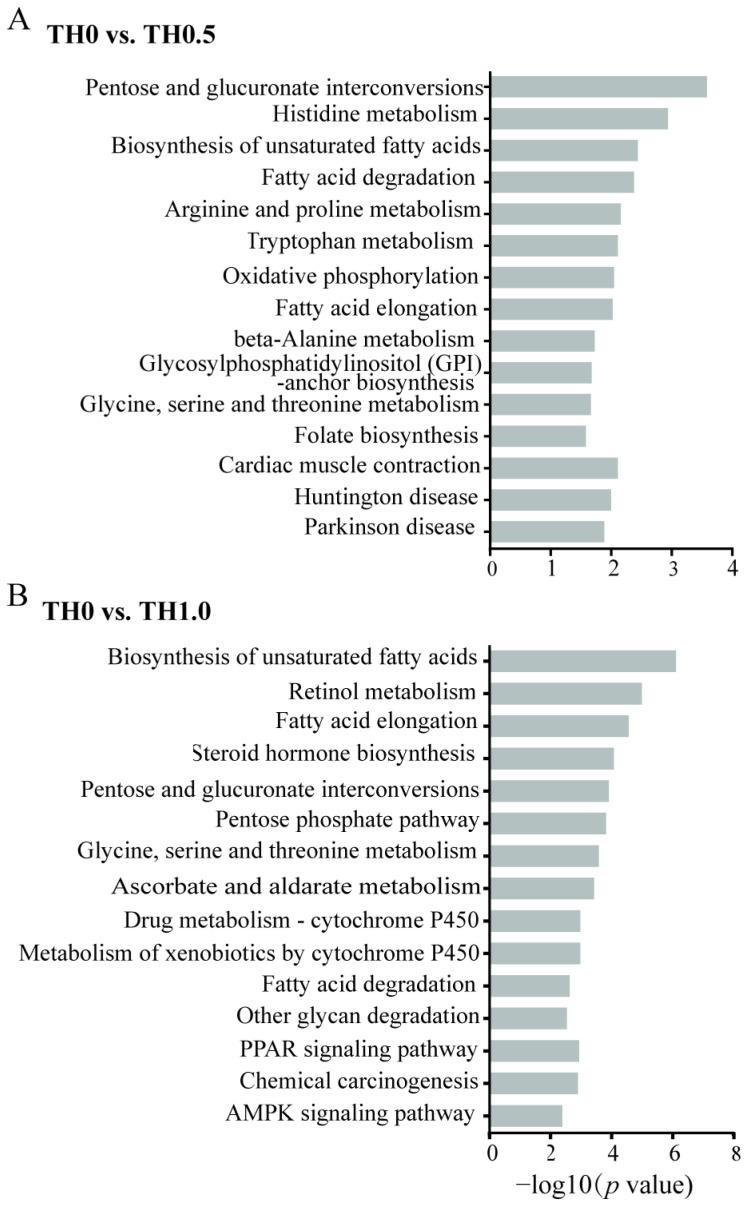
KEGG enrichment analysis of DEGs in the honey bee transcriptome identified from the comparison of the thiacloprid treatment group and the control group. (**A**) KEGG down-enrichment analysis of DEGs in the low concentration group (TH0 vs. TH0.5) and (**B**) the high concentration group (TH0 vs. TH1.0). Vertical axis indicates the pathway name, and the horizontal axis indicates the significance level of DEGs in the secondary classification.

The function of 1140 upregulated and 906 downregulated DEGs were further assessed through GO functional analysis (Figure 5 and Figure S3) and KEGG pathway analysis (Figure 6 and Figure S4). 

Significant GO terms for downregulated DEGs under thiacloprid stress in the early larval stage, as shown in Figure 5, were primarily enriched in the MF (Molecular Function) and BP (Biological Process) categories, including catalytic activity and metabolic process. In the comparison of TH0.5 vs. TH0 shown in Figure 5A, genes that were downregulated were enriched mainly in metabolic-related processes, including membrane lipids, lipids, and sulfur amino acids. Similarly, in the comparison of TH1.0 vs. TH0, the enrichment analysis of downregulated genes indicated a significant association with the lipid metabolic process and fatty acid metabolic process, revealing the impaired metabolism status in the thiacloprid treatment group, which may aggravate honey bee weight loss or even reduce honey bee long-term survival. 

In addition, KEGG pathway enrichment analyses were conducted in the comparison of TH0.5 and TH1.0 groups to controls, as depicted in Figure 6. The results revealed that the AMPK signaling pathway, crucial for memory, was implicated in downstream signaling pathways in TH1.0 (Figure 6B), potentially providing an explanation for the cognitive impairment observed in honey bees exposed to thiacloprid. From the TH0.5 vs. TH0 group, oxidative phosphorylation was enriched in the downregulated DEGs (Figure 6A). The activation of cytochrome P-450 (CYP) is essential for drug metabolism. The pathways related to xenobiotics and drug metabolism by cytochrome P450 were significantly downregulated in the TH1.0 thiacloprid-treated group, such evidence further supports the weakened ability of honey bees to metabolize high thiacloprid concentrations. The apoptosis-related pathway was upregulated in the TH1.0 group (Figure S4B), which is consistent with the fact that thiacloprid treatment induced more apoptotic neurons. 

### 3.6. qRT-PCR Validation Analysis 

**Figure 7:**
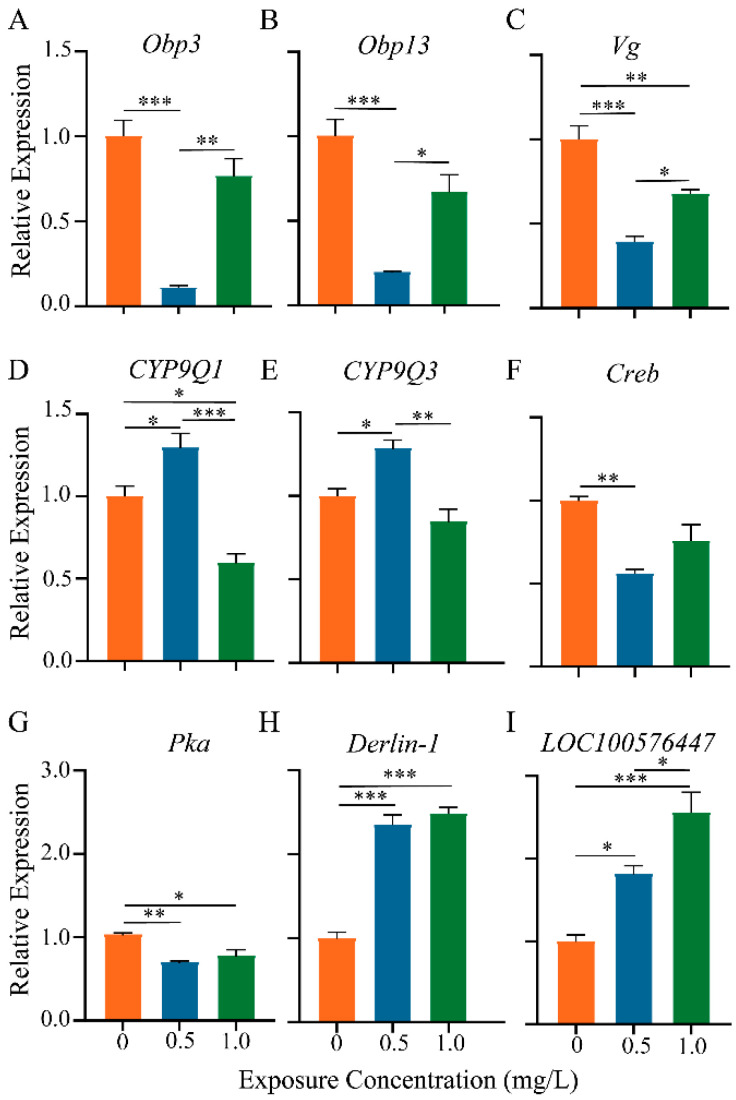
qRT-PCR analysis of relative gene abundance in honey bee brain of each thiacloprid-treated group. The expressions (**A**–**I**) respectively denote the relative expression levels of nine different genes. Results are from three biological replicates of each group and are presented as mean ± SEM. * *p* < 0.05, ** *p* < 0.01, and *** *p* < 0.001.

In Figure 7, nine genes associated with olfactory memory (*Obp3*, *Obp13*, *Creb*, and *Pka*), immune response (*Vg*), apoptosis (*Derlin*-*1* and *LOC100576447*), and detoxification (*CYP9Q1* and *CYP9Q3*) were validated using RT-qPCR. 

The expression patterns of these nine genes were generally in line with our transcriptome sequencing and immunohistochemical findings. Compared to the controls, the level of *Obp3* and *Obp13* expression was reduced in the groups treated with thiacloprid (Figure 7A,B), which could lead to a decrease in olfactory sensitivity. *Creb* and *Pka* are two genes related to learning and memory, the relative expressions of which were also significantly decreased in the thiacloprid treatment groups, as illustrated in Figure 7C,D, which is consistent with the observed decline in the honey bees’ associative olfactory learning and memory tests. The expression of the antioxidant-related gene *Vg* was similarly decreased in the thiacloprid groups. In contrast, the expression of detoxification genes *CYP9Q1* and *CYP9Q3* increased significantly in the groups treated with TH0.5 thiacloprid compared to controls (Figure 7F,G, *p* < 0.05) but decreased in the high-concentration thiacloprid-treated group (TH1.0, Figure 7F). The thiacloprid-treated groups exhibited a significant increase in the apoptosis-associated genes *Derlin*-*1* and *LOC100576447*, as compared to the controls (Figure 7H,I, *Derlin*-*1*: TH1.0 or TH0.5 vs. TH0, *p* < 0.001; *LOC100576447*: TH0.5 vs. TH0, *p* < 0.05, TH1.0 vs. TH0, *p* < 0.001), consistent with the TUNEL staining-mediated apoptosis results.

## 4. Discussion

Honey bee decline is correlated with the use of neonicotinoid insecticides [7]. Thiacloprid, a widely used neonicotinoid, has been found at high field-realistic levels in both pollen and nectar [30]. Studies have shown the adverse effects of thiacloprid on honey bee health [12,17,24], foraging [16], homing [31], social abilities [32], and cognitive behavior [33]. 

Nevertheless, it remains unclear whether the cognitive abilities of adult honey bees will be impaired due to chronic, low-dose exposure to thiacloprid during the larval stage. Here, we assessed the toxicity of thiacloprid exposure during the larval stage using physiological status, cognitive behavioral performance, brain cell apoptosis, and RNA-seq transcriptome analysis. The results indicated that chronic larval exposure to thiacloprid disrupted adult bees’ health and impaired their learning and memory capabilities. Additionally, thiacloprid exposure was found to induce neural apoptosis and alternations in the transcriptome of emerged bees, potentially accounting for the impaired learning and memory behaviors driven by thiacloprid. 

### 4.1. Thiacloprid Disrupted Honey Bee Health

The present study demonstrated a substantial delay in honey bee body weight and a decrease in survival rates when exposed to thiacloprid-induced stress during their larval stage. Moreover, these adverse impacts were more prominent in honey bees subjected to a higher concentration of thiacloprid (TH1.0), indicating that sub-lethal concentrations of thiacloprid inhibited the early physiological development of honey bees from larvae to adulthood. These findings were supported by the earlier investigation that revealed that the delayed development and mortality of honey bees were caused by exposure to neonicotinoids [5,26]. The delayed development and compromised survival can be attributed to factors such as reduced feeding intake [34] and weakened immune function [13]. Although the sensitivity to low-toxic neonicotinoids varied across bee species, our findings align with the prior research, which documented an increase in developmental mortality and prolonged development time in two solitary bee species upon thiacloprid treatment [21].

### 4.2. Thiacloprid Impaired Honey Bee Learning and Memory Ability

Honey bee odor learning and memory ability are important for their foraging action. We showed that chronic larval exposure to thiacloprid impaired honey bee response to sucrose in emerged bees along with olfactory associative learning and memory performance. The results are consistent with previous research that indicated thiacloprid adversely affected honey bee cognitive performance [17,24]. Sucrose sensitivity was used as an important indicator of foraging efficiency. Newly emerged honey bees exposed to thiacloprid treatment at the larval stage exhibited a reduced response to lower concentrations of sucrose (1% and 5%), which is similar to a previous study suggesting that neonicotinoids can lower the sucrose sensitivity of foragers [35]. 

With the exception of sucrose responsiveness, olfactory learning and memory abilities are critical for communication among honey bees within the colony. Our results showed larval thiacloprid exposure affected the learning acquisition and memory retention of emerged bees. Similar adverse effects on learning and memory have been mentioned in previous research on adult bees (honey bee or stingless bees) exposed to thiacloprid [11,16,24]. Furthermore, these findings demonstrated that despite surviving to adulthood, honey bee larvae exposed to thiacloprid experience cognitive disorders after emerging, which aligns with the impaired olfactory associative behavior identified in honey bees exposed to imidacloprid or thiacloprid [17]. 

### 4.3. Larval Exposure to Thiacloprid Inducing Neuronal Apoptosis of Honey Bees

Our immunocytochemistry experiment confirmed neural apoptosis in MB and AL. The apoptotic rates increase significantly in the adult honey bee brain in a dose-dependent manner. Convergent evidence suggested the crucial role of MB and AL in learning and memory encoding in honey bees [36]. Moreover, numerous nAChRs binding sites are widely distributed in the MB and AL. Thiacloprid mimics the action of acetylcholine as an active ingredient, acting on nAChRs with high efficiency. This action leads to the deactivation of neurons, which may be responsible for neuronal apoptosis in these areas. In turn, this could affect the neural encoding of learning and memory within the honey bee brain. Consequently, it may impact honey bee olfactory learning and memory performance. Our findings support recent studies that showed adult honey bee exposure to thiacloprid or imidacloprid led to brain cell apoptosis [24,37]. However, we found that larval exposure to thiacloprid resulted in apoptosis of neuronal cells after the bee emerged, indicating that thiacloprid has a detrimental effect on the development of the honey bee nervous system and, thus, reduced the learning and memory capacity of emerged bees. Exposure to thiacloprid can induce cell apoptosis in the MB and AL of honey bees, potentially leading to a decline in the density of microglomerular organization in the MB, which is crucial for regulating neurotransmitter release. Neurotransmitters are crucial for learning and memory processes in the honey bee brain. Evidence has indicated that a decrease in synapsin levels lead to neurophysiological disorders in MB and AL of the honey bee brain after thiamethoxam exposure [38]. Therefore, the apoptosis detected in MB and AL might be corrected with the low synapsin level and responsible for olfactory functions disorder. 

### 4.4. Thiacloprid Led to Transcriptome Changes in Honey Bees

The expression profile of the brain transcriptome could provide a molecular illustration of the association between neonicotinoid-induced stress, honey bee health, and behavioral change [15,26,39]. Here, functional analysis of DEGs and gene quantification analyses were performed to investigate the impact of larval exposure to thiacloprid on gene expression in adult honey bees’ brains. The findings revealed significant alternations in gene expressions related to metabolic processes, specifically lipid metabolism and histidine metabolism, as well as a decline in catalytic activity. These metabolic changes have the potential to disrupt the normal development of honey bees, as Shi et al.’s work suggested that the brain activity and learning and behavior development of honey bees may be hindered by the reduction of serotonin metabolism caused by thiacloprid [40]. Previous research has highlighted the involvement of odorant-binding proteins (OBPs) in the encoding of olfactory information in honey bees [41]. Our results confirmed the downregulation of *Obp3* and *Obp13* genes, which may explain the adverse effect of thiacloprid on the olfactory sensitivity of honey bees, further affecting honey bee cognition behavior after thiacloprid exposure. Cytochrome P450s, specifically those in the detoxification *CYP9Q* subfamily, have been found to influence the sensitivity of bees to neonicotinoids. *CYP9Q*3 has been shown to efficiently metabolize thiacloprid [42]. Our transcriptome sequencing results revealed that both *CYP9Q1* and *CYP9Q3* were downregulated following thiacloprid treatment (Figure 4 and Figure 7D,E), which aligns with previous research findings [5]. Yet, another study indicated that clothianidin did not affect the expression of detoxification enzymes *CYP9Q* [43]. This suggests that different subfamilies of *CYP9Q* may be involved in the detoxification of different neonicotinoids. Further investigations are necessary to definitively determine how the *CYP9Q* subfamily enhances resistance to thiacloprid and clarify the potential variations in detoxification mechanisms across neonicotinoid compounds. In addition, we detected a high expression of two apoptosis-related genes *Derlin*-*1* and *LOC1005764470*, which is consistent with the apoptotic damage of MB and AL neurons (Figure 3). These results align with previous studies [24,37] that implied the induction of apoptosis in brain cells following treatment with imidacloprid or thiacloprid in adult bees. How are the transcriptomic alterations associated with changes in behaviors during neonicotinoids toxicity conditions? Functional analysis of DEGs revealed alternations in the cAMP and AMPK signaling pathways, both of which have been implicated in learning and memory processes [44,45]. These findings suggest that cognitive disorder after thiacloprid exposure could be attributed to the dysregulation of these signaling pathways. Moreover, we identified the decreased expression of genes related to learning and memory, *Creb* and *Pka* (Figure 7F,G), further supporting the connection between thiacloprid exposure and honey bee learning and memory impairment. These results are similar to an earlier study that reported the downregulation of *Pka* and *Creb* genes after neonicotinoid exposure in adult bees [24,46].

## 5. Conclusions

In conclusion, chronic exposure of honey bee larvae to low-dose thiacloprid has significant impacts on the physiological state and cognitive abilities of adult honey bees. This is the initial report documenting that chronic larval exposure to thiacloprid induced neural apoptosis in the mushroom bodies (MB) and antennal lobes (AL) of adult bees, accompanied by transcriptome alterations in genes (*Obp3*, *Obp13*, *Creb*, *Pka*, *Vg*, *Derlin*-*1*, *LOC100576447*, *CYP9Q1*, and *CYP9Q3*) and pathways associated with learning and memory. These findings suggested a potential link between learning and memory disorders and the neuronal and molecular changes induced by thiacloprid. The negative impacts of larval thiacloprid exposure are long-lasting and chronic, persisting from larval development to adulthood and potentially extending to the health and productivity of honey bee populations. Our results emphasized the necessity for increased attention and careful use of neonicotinoid insecticides, even when their toxicity is considered low, to safeguard honey bee populations and promote their well-being.

## Data Availability

RNA-seq raw data were submitted to the NCBI Bioproject database (Bioproject ID: PRJNA929684).

## Supplementary Materials

The following supporting information can be downloaded at https://www.mdpi.com/article/10.3390/toxics12010018/s1, Figure S1: Apoptosis of cells in the internal chiasma (IC), outer optic chiasma (OC), and Lamina (LA) in honey bee brain treated with thiacloprid; Figure S2: Principal component analysis (PCA) plot and Heatmap of transcriptome data; Figure S3: Up-Enrichment GO term in groups of TH0 vs. TH0.5 and TH0 vs. TH1.0; Figure S4: Up-Enrichment KEGG term in groups of TH0 vs. TH0.5 and TH0 vs. TH1.0; Table S1: Composition of feed formulas used in larva rearing; Table S2: Detailed information of All primers (5′-3′) for qRT-PCR used in this study.

## References

[1] Cilia G., Flaminio S., Zavatta L., Ranalli R., Quaranta M., Bortolotti L., Nanetti A. (2022). Occurrence of honey bee (*Apis mellifera* L.) pathogens in wild pollinators in northern Italy. Front. Cell Infect. Microbiol..

[2] Matthijs S., De Waele V., Vandenberge V., Verhoeven B., Evers J., Brunain M., Saegerman C., De Winter P.J.J., Roels S., de Graaf D.C. (2020). Nationwide screening for bee viruses and parasites in belgian honey bees. Viruses.

[3] Wang K., Chen H., Fan R.L., Lin Z.G., Niu Q.S., Wang Z., Ji T. (2022). Effect of carbendazim on honey bee health: Assessment of survival, pollen consumption, and gut microbiome composition. Ecotoxicol. Environ. Saf..

[4] Branchiccela B., Castelli L., Corona M., Diaz-Cetti S., Invernizzi C., de la Escalera G.M., Mendoza Y., Santos E., Silva C., Zunino P. (2019). Impact of nutritional stress on the honeybee colony health. Sci. Rep..

[5] Zhao H., Li G.L., Cui X.P., Wang H.F., Liu Z.G., Yang Y.W., Xu B.H. (2022). Review on effects of some insecticides on honey bee health. Pestic. Biochem. Phys..

[6] Matsuda K., Ihara M., Sattelle D.B. (2020). Neonicotinoid insecticides: Molecular targets, resistance, and toxicity. Annu. Rev. Pharmacol. Toxicol..

[7] Woodcock B.A., Bullock J.M., Shore R.F., Heard M.S., Pereira M.G., Redhead J., Ridding L., Dean H., Sleep D., Henrys P. (2017). Country-specific effects of neonicotinoid pesticides on honey bees and wild bees. Science.

[8] Wang X., Goulson D., Chen L., Zhang J., Zhao W., Jin Y., Yang S., Li Y., Zhou J. (2020). Occurrence of neonicotinoids in chinese apiculture and a corresponding risk exposure assessment. Environ. Sci. Technol..

[9] Kavanagh S., Henry M., Stout J.C., White B. (2021). Neonicotinoid residues in honey from urban and rural environments. Environ. Sci. Pollut. Res. Int..

[10] Purdy J. (2018). Distribution of residues of neonicotinoids in the hive and in bees in relation to bee health. Jul.-Kuhn-Arch..

[11] Jacob C.R.O., Malaquias J.B., Zanardi O.Z., Silva C.A.S., Jacob J.F.O., Yamamoto P.T. (2019). Oral acute toxicity and impact of neonicotinoids on *Apis mellifera* L. and *Scaptotrigona postica* latreille (Hymenoptera: Apidae). Ecotoxicology.

[12] Lv L., Li W., Li X., Wang D., Weng H., Zhu Y.-C., Wang Y. (2023). Mixture toxic effects of thiacloprid and cyproconazole on honey bees (*Apis mellifera* L.). Sci. Total Environ..

[13] Brandt A., Gorenflo A., Siede R., Meixner M., Buchler R. (2016). The neonicotinoids thiacloprid, imidacloprid, and clothianidin affect the immunocompetence of honey bees (*Apis mellifera* L.). J. Insect Physiol..

[14] Liu Y.J., Qiao N.H., Diao Q.Y., Jing Z.W., Vukanti R., Dai P.L., Ge Y. (2020). Thiacloprid exposure perturbs the gut microbiota and reduces the survival status in honeybees. J. Hazard. Mater..

[15] Fent K., Schmid M., Hettich T., Schmid S. (2020). The neonicotinoid thiacloprid causes transcriptional alteration of genes associated with mitochondria at environmental concentrations in honey bees. Environ. Pollut..

[16] Tison L., Hahn M.L., Holtz S., Rossner A., Greggers U., Bischoff G., Menzel R. (2016). Honey bees’ behavior is impaired by chronic exposure to the neonicotinoid thiacloprid in the field. Environ. Sci. Technol..

[17] Ke L., Chen X., Dai P., Liu Y.-J. (2023). Chronic larval exposure to thiacloprid impairs honeybee antennal selectivity, learning and memory performances. Front. Physiol..

[18] Sanchez-Bayo F., Goka K. (2014). Pesticide residues and bees—A risk assessment. PLoS ONE.

[19] Stuligross C., Williams N.M. (2021). Past insecticide exposure reduces bee reproduction and population growth rate. Proc. Natl. Acad. Sci. USA.

[20] Shi J., Zhang R., Pei Y., Liao C., Wu X. (2020). Exposure to acetamiprid influences the development and survival ability of worker bees (*Apis mellifera* L.) from larvae to adults. Environ. Pollut..

[21] Claus G., Pisman M., Spanoghe P., Smagghe G., Eeraerts M. (2021). Larval oral exposure to thiacloprid: Dose-response toxicity testing in solitary bees, *Osmia* spp. (Hymenoptera: Megachilidae). Ecotoxicol. Environ. Saf..

[22] Friol P.S., Catae A.F., Tavares D.A., Malaspina O., Roat T.C. (2017). Can the exposure of *Apis mellifera* (Hymenoptera, Apiadae) larvae to a field concentration of thiamethoxam affect newly emerged bees?. Chemosphere.

[23] Tison L., Holtz S., Adeoye A., Kalkan O., Irmisch N.S., Lehmann N., Menzel R. (2017). Effects of sublethal doses of thiacloprid and its formulation Calypso (R) on the learning and memory performance of honey bees. J. Exp. Biol..

[24] Li A., Yin L., Ke L., Diao Q.-Y., Wu Y., Dai P., Liu Y.-J. (2023). Thiacloprid impairs honeybee worker learning and memory with inducing neuronal apoptosis and downregulating memory-related genes. Sci. Total Environ..

[25] Schmehl D.R., Tome H.V.V., Mortensen A.N., Martins G.F., Ellis J.D. (2016). Protocol for the in vitro rearing of honey bee (*Apis mellifera* L.) workers. J. Apic. Res..

[26] Li B., Ke L., Li A.-R., Diao Q.Y., Wang Q., Liu Y.J. (2022). Exposure of larvae to sublethal thiacloprid delays bee development and affects transcriptional responses of newly emerged honey bees. Front. Insect Sci..

[27] Pohorecka K., Skubida P., Miszczak A., Semkiw P., Sikorski P., Zagibajlo K., Teper D., Koltowski Z., Skubida M., Zdanska D. (2012). Residues of neonicotinoid insecticides in bee collected Plant materials from oilseed rape crops and their effect on bee colonies. J. Apic. Sci..

[28] Ebeling J., Pieper F., Gobel J., Knispel H., McCarthy M., Goncalves M., Turner M., Rod Merrill A., Genersch E. (2021). Anti-Virulence strategy against the honey bee pathogenic bacterium *Paenibacillus larvae* via small molecule inhibitors of the bacterial toxin Plx2A. Toxins.

[29] Denton J.A., Koludarov I., Thompson M., Bryk J., Velasque M. (2021). Honey bee cognition as a tool for scientific engagement. Insects.

[30] Zioga E., Kelly R., White B., Stout J.C. (2020). Plant protection product residues in plant pollen and nectar: A review of current knowledge. Environ. Res..

[31] Christen V., Grossar D., Charrière J.-D., Eyer M., Jeker L. (2021). Correlation between increased homing flight duration and altered gene expression in the brain of honey bee foragers after acute oral exposure to thiacloprid and thiamethoxam. Front. Insect Sci..

[32] Forfert N., Moritz R.F.A. (2017). Thiacloprid alters social interactions among honey bee workers (*Apis mellifera*). J. Apic. Res..

[33] Tison L., Rossner A., Gerschewski S., Menzel R. (2019). The neonicotinoid clothianidin impairs memory processing in honey bees. Ecotoxicol. Environ. Saf..

[34] Ohlinger B.D., Schurch R., Durzi S., Kietzman P.M., Silliman M.R., Couvillon M.J. (2022). Honey bees (Hymenoptera: Apidae) decrease foraging but not recruitment after neonicotinoid exposure. J. Insect Sci..

[35] Demares F.J., Pirk C.W.W., Nicolson S.W., Human H. (2018). Neonicotinoids decrease sucrose responsiveness of honey bees at first contact. J. Insect Physiol..

[36] Andrione M., Vallortigara G., Antolini R., Haase A. (2016). Neonicotinoid-induced impairment of odour coding in the honeybee. Sci. Rep..

[37] Wu Y.Y., Zhou T., Wang Q., Dai P.L., Xu S.F., Jia H.R., Wang X. (2015). Programmed cell death in the honey bee (*Apis mellifera*) (Hymenoptera: Apidae) worker brain induced by imidacloprid. J. Econ. Entomol..

[38] Tavares D.A., Roat T.C., Silva-Zacarin E.C.M., Nocelli R.C.F., Malaspina O. (2019). Exposure to thiamethoxam during the larval phase affects synapsin levels in the brain of the honey bee. Ecotoxicol. Environ. Saf..

[39] Li Z., Yu T., Chen Y., Heerman M., He J., Huang J., Nie H., Su S. (2019). Brain transcriptome of honey bees (*Apis mellifera*) exhibiting impaired olfactory learning induced by a sublethal dose of imidacloprid. Pestic. Biochem. Physiol..

[40] Shi T.F., Burton S., Wang Y.F., Xu S.Y., Zhang W.X., Yu L.S. (2018). Metabolomic analysis of honey bee, *Apis mellifera* L. response to thiacloprid. Pestic. Biochem. Physiol..

[41] Foret S., Maleszka R. (2006). Function and evolution of a gene family encoding odorant binding-like proteins in a social insect, the honey bee (*Apis mellifera*). Genome Res..

[42] Manjon C., Troczka B.J., Zaworra M., Beadle K., Randall E., Hertlein G., Singh K.S., Zimmer C.T., Homem R.A., Lueke B. (2018). Unravelling the molecular determinants of bee sensitivity to neonicotinoid insecticides. Curr. Biol..

[43] Tsvetkov N., Bahia S., Calla B., Berenbaum M.R., Zayed A. (2023). Genetics of tolerance in honeybees to the neonicotinoid clothianidin. iScience.

[44] Fiala A., Muller U., Menzel R. (1999). Reversible downregulation of protein kinase a during olfactory learning using antisense technique impairs long-term memory formation in the honeybee, *Apis mellifera*. J. Neurosci..

[45] Finley J. (2018). Facilitation of hippocampal long-term potentiation and reactivation of latent HIV-1 via AMPK activation: Common mechanism of action linking learning, memory, and the potential eradication of HIV-1. Med. Hypotheses.

[46] Christen V., Mittner F., Fent K. (2016). Molecular effects of neonicotinoids in honey bees (*Apis mellifera*). Environ. Sci. Technol..

