# Supplementary material for: Early-Life Sublethal Thiacloprid Exposure to Honey Bee Larvae: Enduring Effects on Adult Bee Cognitive Abilities
Source: Toxics. 2023 Dec 23;12(1):18. doi: 10.3390/toxics12010018 (PMC10820931; doi:10.3390/toxics12010018)
Supplement: Supplementary file 1 [file toxics-12-00018-s001.zip › toxics-2750956-supplementary.pdf]

toxics

Supporting information

Chronic thiacloprid exposure to honey bee larvae at environmental concentration disrupts adult bee cognitive abilities

Xiasang Chen, Airui Li, Linghong Yin, Li Ke, Pingli Dai and Yong-Jun Liu*

State Key Laboratory of Resource Insects, Institute of Apicultural Research, Chinese Academy of Agricultural Sciences, Beijing, 100193, China

Contents

Results

Tables

Table S1. Composition of feed formulas used in larva rearing.

Table S2. Detailed information of All primers (5′-3′) for qRT-PCR used in this study.

Figures:

S1. Apoptosis of cells in the internal chiasma (IC), outer optic chiasma (OC), and Lamina (LA) in honey bee brain treated with thiacloprid.

S2. Principal component analysis (PCA) plot and Heatmap of transcriptome data.

S3. Up-Enrichment GO term in groups of TH0 vs. TH0.5 and TH0 vs. TH1.0.

S4. Up-Enrichment KEGG term in groups of TH0 vs. TH0.5 and TH0 vs. TH1.0.

Results

Upregulated DEGs functional analysis

Significant GO terms for upregulated DEGs under thiacloprid stress in the early larval stage, as shown in FigureS3, were mainly in the MF (Molecular Function) and CC (Cellular Component) categories, specifically related to components of membrane and extracellular region. Based on the upregulated DEGs, the thiacloprid treatment group with TH0.5 exhibited enrichment GO terms of catalase activity and peroxidase activity (FigureS3A). Similarly, the thiacloprid treatment group with TH1.0 displayed significant GO enrichment in the defense response, in comparison to the control (FigureS3B). These results suggested that exposure to thiacloprid may promote peroxide resistance and immune response in honey bees.

In addition, KEGG pathway enrichment results revealed that the Dopaminergic synapse and cAMP signaling pathways, which are critical pathways involved in learning and memory, were enriched in the up-regulated DEGs between thiacloprid-treated groups of TH0 and TH0.5 (FigureS4A). It may contribute to the cognitive disorder of honey bees caused by thiacloprid. The pathway of apoptosis was also upregulated in the group of TH1.0 (FigureS4B).

Figure S1. Apoptosis of cells in the internal chiasma (IC), outer optic chiasma (OC), and Lamina (LA) in

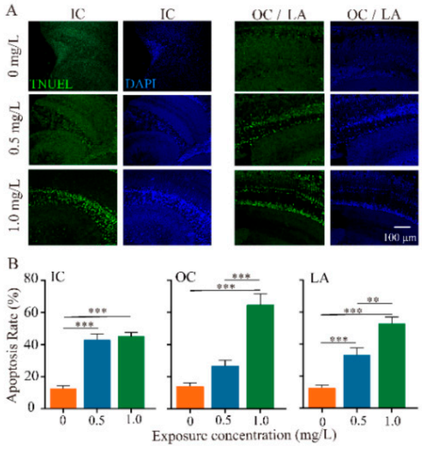

honey bee brain treated with thiacloprid. Representative images of apoptosis in IC, OC, and LA identified by TUNEL staining. (B). Quantification of apoptosis cells in three brain regions of honey bees (IC, OC, and LA) in each treatment group. Bars are mean ± SEM. *p < 0.05, **p < 0.01 and ***p < 0.001.

Figure S2. (A). Principal component analysis (PCA) plot of transcriptomes gene expression. (B) Heatmap of

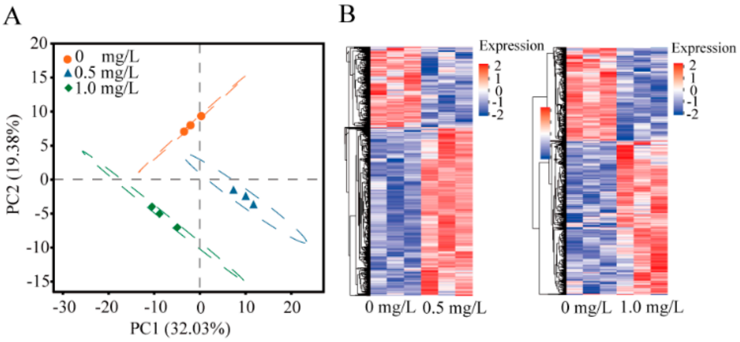

gene expression of transcriptome data of honey bees among each treatment group. Each column represents a sample and each row corresponds to a gene. The color scale indicates the normalized expression values of the genes in each sample, with red indicating high expression and blue indicating low expression.

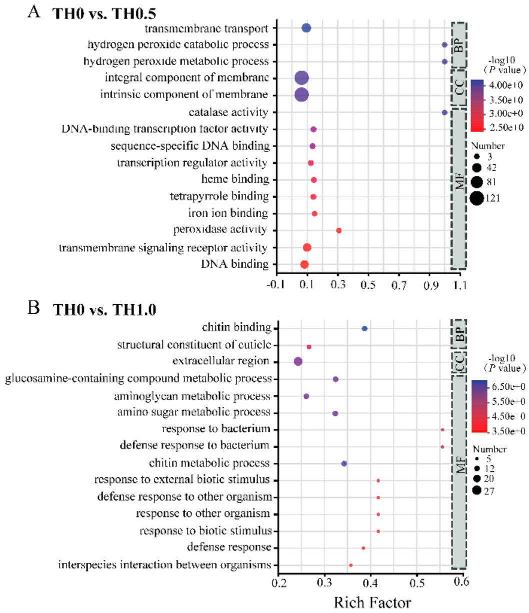

Figure S3. (A). GO-up-enrichment analysis of DEGs in low concentration group (TH0 vs. TH0.5) and (B). in high concentration group (TH0 vs. TH1.0). The vertical axis represents the secondary classification terms of GO, while the horizontal axis represents the Rich factor. The size and color of the dots correspond to the number of genes and the -log10 (*P* value) ranges, respectively.

Figure S4. (A). KEGG-up-enrichment analysis of DEGs in low concentration group (TH0 vs. TH0.5) and (B).

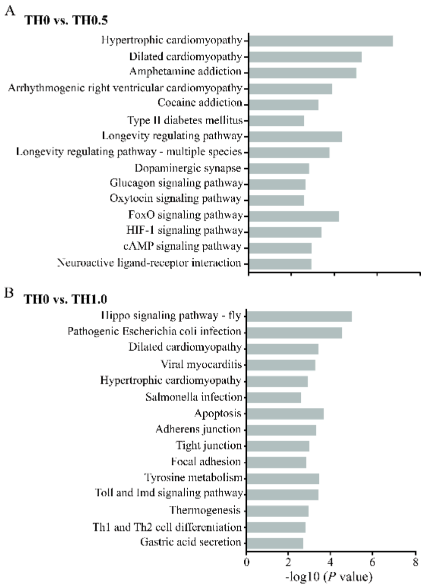

in high concentration group (TH0 vs. TH1.0). Vertical axis indicates the pathway name and the horizontal axis is the significance level of DEGs in the secondary classification.

Table S1. Composition of feed formulas used in larva rearing.

Types of food	Feed formulas					Date of feeding	Feeding volume of each larva (μL)
	Glucose (g)	Fructose (g)	Yeast (g)	Royal jelly (g)	Sterile water (mL)		
A	5.3	5.3	0.9	44.25	44.25	D1	20
B	6.4	6.4	1.3	42.95	42.95	D2-D3	20
C	9	9	2	50	30	D4-D6	30/40/50

Table S2 Detailed information of All primers (5′-3′) for qRT-PCR used in this study.

Genes	All sequences showed 5′ to 3′		Accession number
	Forward	Reverse	
β-actin	TGCCAACACTGTCCTTTCTG	AGAATTGACCCACCAATCCA	NM_001185146.1
Obp3	ATGATGGTTCGTTGTGACGA	GCACTTATCCTCGTTCTTAGCA	NM_001040221.1
Obp13	ATAGAGTCAGTTTGCGCCGA	CACTGTCTAATTTCGTTGTCC	NM_001040224.1
LOC409791 (*Pka*)	AAGATTGTTAGCGGAAAGCCAC	GACCCTTGATATCGTTTACACCAG	XM_393285.7
LOC410492 (*CYP9Q1*)	ACTCGAGAGGGGGATGAACA	AAACCTGTCCGGGTCGAATC	XM_006562301.3
LOC727262 (*Derlin-1*)	TGTTAATTTCTGGTTTGGTACATCA	AGTTCCATCATACCACCTCCA	XM_001122972.5
Vg	ACGCATCACGAATACGACTACG	CTCAGGCTCAACTCCATGAAAG	NM-001011578
LOC408453 (*CYP9Q1*)	GTAGGGAGAAGTTGGGCACC	GTCTCGTCGATCTCCTGCTG	XM_006562300.3
LOC10057647	AGAATGGGATCAGACGGTGC	TGTCCCAGAGCACTCGAGAA	NM_001278338.2
LOC409401 （*Creb*）	TCTCCAGAACAAAGAGGAGGTATC	ATGAAGCGAATGGGAAGACAC	XM_006570049.3
